# Blood Compatibility of Amphiphilic Phosphorous Dendrons—Prospective Drug Nanocarriers †

**DOI:** 10.3390/biomedicines9111672

**Published:** 2021-11-12

**Authors:** Simon Suty, Veronika Oravczova, Zuzana Garaiova, Veronika Subjakova, Maksim Ionov, Dzmitry Shcharbin, Zuzana Simonikova, Peter Bartek, Milan Zvarik, Xiangyang Shi, Serge Mignani, Jean-Pierre Majoral, Maria Bryszewska, Tibor Hianik, Iveta Waczulikova

**Affiliations:** 1 Department of Nuclear Physics and Biophysics, Faculty of Mathematics, Physics and Informatics, Comenius University, Mlynska Dolina F1, 84248 Bratislava, Slovakia; simon.suty@fmph.uniba.sk (S.S.); Oravczova.V@gmail.com (V.O.); zuzana.garaiova@fmph.uniba.sk (Z.G.); veronika.subjakova@fmph.uniba.sk (V.S.); milan.zvarik@fmph.uniba.sk (M.Z.); tibor.hianik@fmph.uniba.sk (T.H.); 2 Department of General Biophysics, Faculty of Biology and Environmental protection, University of Lodz, 141/143 Pomorska St., 90-236 Lodz, Poland; maksim.ionov@biol.uni.lodz.pl (M.I.); maria.bryszewska@biol.uni.lodz.pl (M.B.); 3 Institute of Biophysics and Cell Engineering, National Academy of Sciences of Belarus (IBCENASB), Akademicheskaya 27, 220072 Minsk, Belarus; d.shcharbin@gmail.com; 4 Department of Hematology and Transfusiology, St. Elizabeth Cancer Institute, Heydukova 10, 81250 Bratislava, Slovakia; zuzana.simonikova@ousa.sk (Z.S.); peter.bartek@ousa.sk (P.B.); 5 CQM—Centro de Química da Madeira, MMRG, Universidade da Madeira, Campus da Penteada, 9020-105 Funchal, Portugal; xshi@dhu.edu.cn (X.S.); serge.mignani@parisdescartes.fr (S.M.); 6 State Key Laboratory for Modification of Chemical Fibers and Polymer Materials, College of Chemistry, Chemical Engineering and Biotechnology, Donghua University, Shanghai 201620, China; 7 Laboratoire de Chimie et de Biochimie Pharmacologiques et Toxicologique, Université Paris Descartes, PRES Sorbonne Paris Cité, CNRS UMR 860, 45 Rue des Saints Peres, 75006 Paris, France; 8 Laboratoire de Chimie de Coordination du CNRS, 205 Route de Narbonne, CEDEX 4, 31077 Toulouse, France; jean-pierre.majoral@lcc-toulouse.fr; 9 Université Toulouse, 118 Route de Narbonne, CEDEX 4, 31077 Toulouse, France

**Keywords:** nanoparticles, amphiphilic dendrons, blood compatibility, liposomes

## Abstract

Dendrons are branched synthetic polymers suitable for preparation of nanosized drug delivery systems. Their interactions with biological systems are mainly predetermined by their chemical structure, terminal groups, surface charge, and the number of branched layers (generation). Any new compound intended to be used, alone or in combination, for medical purposes in humans must be compatible with blood. This study combined results from in vitro experiments on human blood and from laboratory experiments designed to assess the effect of amphiphilic phosphorous dendrons on blood components and model membranes, and to examine the presence and nature of interactions leading to a potential safety concern. The changes in hematological and coagulation parameters upon the addition of dendrons in the concentration range of 2–10 µM were monitored. We found that only the combination of higher concentration and higher generation of the dendron affected the selected clinically relevant parameters: it significantly decreased platelet count and plateletcrit, shortened thrombin time, and increased activated partial thromboplastin time. At the same time, occasional small-sized platelet clumps in blood films under the light microscope were observed. We further investigated aggregation propensity of the positively charged dendrons in model conditions using zwitterionic and negatively charged liposomes. The observed changes in size and zeta potential indicated the electrostatic nature of the interaction. Overall, we proved that the low-generation amphiphilic phosphorous dendrons were compatible with blood within the studied concentration range. However, interactions between high-generation dendrons at bulk concentrations above 10 µM and platelets and/or clotting factors cannot be excluded.

## 1. Introduction

Dendrons are synthetic molecules, a constituting subunit of larger, nanosized supramolecular structures—dendrimers. Dendrimers are widely accepted as a fourth class of polymers [1]. The size of dendrons and dendrimers is dependent on the number of repetition cycles, called generations (G). Based on their unique structure and composition, the dendritic nanoparticles (NPs) are widely studied in various areas, including medical applications; e.g., as drug carriers in cancer treatment or antimicrobial therapy. The drug molecules can be entrapped within their inner cavities or chemically conjugated on their surface [2,3,4,5]. A new concept of forming dendron corona at virus surfaces was reported for attaching the cell-targeting groups/drug molecules to improve the biodistribution and capacity in virus-assisted gene therapy [6].

In targeted drug delivery, the first tissue that NP carriers encounter is usually blood (blood is considered a connective tissue because it has a matrix.) Therefore, compatibility with blood and its elements is a necessary requirement for the application of NPs in vivo and especially their interactions with platelets and the enzymes of the coagulation cascade, since these play a central role in hemostasis and its regulation [7,8]. The potential incompatibility of nanomaterials with platelets remains to be completely understood. In this sense, hemocompatibility of NP is a crucial factor in hindering undesired activation of elements of the coagulation cascade and the following blood coagulation and thrombosis, which could even lead to emboli [9]. Due to the substantial role of platelets in thrombosis, the study of interactions between NP and platelets (activation or inhibition) might define their thrombogenicity and biocompatibility [10].

Several studies have reported that the surface chemistry of NPs is one of the most underlying factors by which NPs can activate platelets [11]. Studies performed by Dobrovolskaia et al. using 12 different formulations of PAMAM dendrimers with diverse sizes and surface charges (G3, G4, G5, and G6 functionalized with succinamic acid (anionic), amine (cationic) and amidoethanol groups (neutral)) on human platelets showed that only large (G4–G6) cationic dendrimers, but not the small (G3) cationic or anionic or neutral dendrimers, exhibited collagen-induced platelet aggregation [12]. Between different nanoplatforms, cationic dendrimers noticeably showed their effect on thrombocyte activation through inducing aggregation of platelets in vitro and in vivo. Cationic PAMAM dendrimers (with amine end-group) G7 may cause coagulopathies through aggregation of negatively charged blood proteins such as fibrinogen and albumin [13]. It has been reported that PAMAM dendrimers activate platelets and dramatically alter their morphology [14,15]. The activation/inhibition of platelets through electrostatic interaction with NP is a controversial phenomenon that should be studied in each case separately, because these effects are also dependent on different physico-chemical properties such as size, shape, and chemical composition of the NPs [16].

In vitro assays using human blood along with experiments using cell models appears to be a suitable technique to study mechanisms of dendron-based nanomedicines’ interference with hemostasis and to optimize their hemocompatibility. Therefore, in this work, we have focused on the interactions of amphiphilic phosphorous dendrons of two generations with model lipid membrane—liposomes and with whole human blood to ascertain whether and how these dendrons affect the blood elements, as well as the coagulation pathway.

## 2. Materials, Subjects and Methods

### 2.1. Chemicals and Dendrons

**Figure A1:**
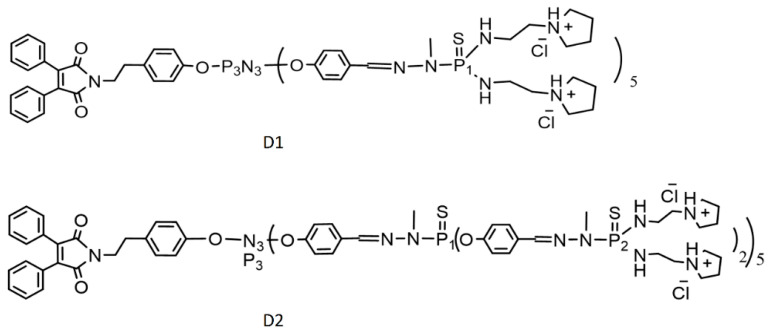
Chemical structure of the studied amphiphilic dendrons of the first (D1) and second (D2) generation. Details on the preparation of D1 and D2 have been published as supplementary data to the article [17], where dendron D1 is denoted as D1G1·HCl and dendron D2 as D1G2·HCl. This supporting information is available free of charge at https://pubs.acs.org/doi/10.1021/acs.bioconjchem.0c00716 (accessed on 4 November 2021).

We used 10 mM phosphate buffer, pH 7.4, adjusted using 1M HCl or 1M NaOH at a temperature of 25 °C. 1,2-dimyristoyl-sn-glycero-3-phosphocholine (DMPC), 1,2-dimyristoyl-sn-glycero-3-phospho-rac-(1-glycerolu) sodium salt (DMPG) and cholesterol (Chol) lipids were purchased from Sigma Aldrich (Darmstadt, Germany). In this work, amphiphilic phosphorus dendrons of the first (D1) and second (D2) generation were prepared according to [17] and studied. The chemical structures and molar weights were: C124H198Cl10N34O8P8S5, Mw = 3055.77 g/mol; and C264H418Cl20N74O18P18S15, Mw = 6664.18 g/mol, respectively (Figure A1).

### 2.2. Preparation of Liposomes

We used liposomes as a simple model of cell membranes. For the composition of liposomes, we used a mixture of DMPG, DMPC and cholesterol (DMPG-DMPC-Chol) in the respective ratios. DMPG represented an amount of 10 mol% from the DMPC, and Chol was added in an amount of 30 mol% of the mixture of DMPC and DMPG. The dry lipid film was hydrated with 10 mM Na-phosphate buffer, pH 7.4, resulting in 10 mg/mL of the lipids in the solution. The liposomes were extruded through a polycarbonate membrane using mini-extruder set with a heating block (Avanti Polar Lipids Inc., Alabaster, AL, USA) with a 400 nm pore size to obtain unilamellar liposomes.

### 2.3. Size and Zeta Potential Measurements

The size of the prepared liposomes upon the interactions with D1 and D2 dendrons was measured by a dynamic light-scattering technique (DLS), and Zeta potential was measured by a phase analysis light-scattering technique based on laser Doppler velocimetry using a Zetasizer-Nano ZS90 spectrophotometer (Malvern Instrument, Malvern, UK). The concentration of lipid vesicles was 1 mg/mL. A stock solution of dendrons dissolved in Milli-Q water at a concentration of 3 mM was prepared. Dendrons were titrated into the liposomal solution by adding small aliquots from one of three working solutions. The first was a 0.05 mM solution used for a final dendron concentration of 0.1 µM; the second was a 0.5 mM solution used for preparing samples with final concentration of 1 µM; and the last was a 1.5 mM solution used for preparing higher final dendron concentrations (3 µM, 4 µM, 5 µM, 7.5 µM, 10 µM, 12.5 µM, and 15 µM). Z-average size—the hydrodynamic size parameter (also known as the cumulants mean), and width parameter of that intensity-weighted size distribution, known as the polydispersity index (PDI) were analyzed using Malvern software. Zeta potential measurements were performed in a folded capillary cell (DTS1070) (Malvern Panalytical Inc., Westborough, MA, USA). All measurements were performed independently at 25 °C and at 37 °C.

### 2.4. Blood Compatibility Study

Fasting blood specimens were collected from healthy volunteers in the morning. To control for age, sex, and other potential confounders, we used a fully within-subject study design, including balanced numbers of male (*n* = 7) and female (*n* = 7) participants aged between 20 and 28 years. This study was conducted in accordance with the ethical principles set forth in the Declaration of Helsinki and the study protocol was approved by the Institutional Ethics Committee at the St. Elizabeth Cancer Institute in Bratislava (Approval No. 03-2020/EK OÚSA, signed on 4 March 2020).

For the investigation of hematological parameters, we used the COULTER DxH 800 automatic hematology analyzer (Beckman Coulter Inc., Brea, CA, USA); and for coagulation parameters, we used the ACL Top 500 CTS automatic hemostasis analyzer (Instrumentation Laboratory, Bedford, MA, USA). The blood was taken by blood vacutainers containing sodium citrate for investigating coagulation parameters and vacutainers containing K_2_EDTA for investigating blood elements. Both generations, D1 and D2, were tested in vitro at each of the selected final dendron concentrations, 2 µM (C1) and 10 µM (C2). The limits of the concentration range were selected based on preliminary results of cytotoxicity studies using several cell lines, a hemolysis study, and a study performed on platelet-rich plasma samples with an increasing final concentration of dendrons (0, 1, 5, 10, and 20 µM). In this study, we prepared the blood samples as follows: the vacutainer tubes were gently mixed, and 1 mL aliquots of blood were transferred to six Eppendorf tubes. Then, either 4 µL or 20 µL from a respective dendron stock solution at 0.5 mM was added to reach the final dendron concentrations of 2 µM (C1) and 10 µM (C2), respectively. The control samples were supplemented with 20 µL of 0.9% NaCl intravenous solution. Two control samples for each measurement series were prepared. The first one was processed immediately, and the second one was incubated together with other samples for three hours at a room temperature, protected from light. All samples were manually stirred at regular intervals during the incubation. Hematological investigation was completed with examination of fixed and stained blood smears (films) of each sample. All samples were viewed and described by an experienced hematologist.

### 2.5. Statistical Analysis

The collected experimental and clinical two-factor laboratory data were summarized using descriptive statistics. Continuous variables are presented as means with the respective SD (standard deviation). The degree of departure from normality was assessed with Shapiro–Wilk test. Simple bivariate correlations (Pearson’s parametric or Spearman’s nonparametric) for all pairwise combinations of the variables were computed to indicate existence and degree of mutual relationships. Analysis of variance (ANOVA) for two-factor fully within-subject design was performed to assess effects of both factors (concentration and generation), as well as their interaction (concentration × generation). The restricted maximum likelihood approach for estimating components of variance was used to control for nuisance variables, such as sex and fitness. The estimated effect sizes in the form of differences are presented along with the respective 95% confidence intervals (95% CI).

For analysis of particle size and zeta potential measurements, the dependence of zeta-average size and zeta potential on the dendron concentration at the given experimental condition was recorded, and the resulting curves were compared using the extra sum-of-squares F test and the corrected Akaike’s criterion (AICc).

Statistical analyses were performed using StatsDirect 3.3.5 software (Stats Direct Ltd., Cheshire, UK) and GraphPad Prism 9.0 (GraphPad Software Inc., San Diego, CA, USA). All *p*-values were considered significant at a two-tailed *p*-value of <0.05.

## 3. Results

### 3.1. Dendrons’ Blood Compatibility Assessment

**Figure 1:**
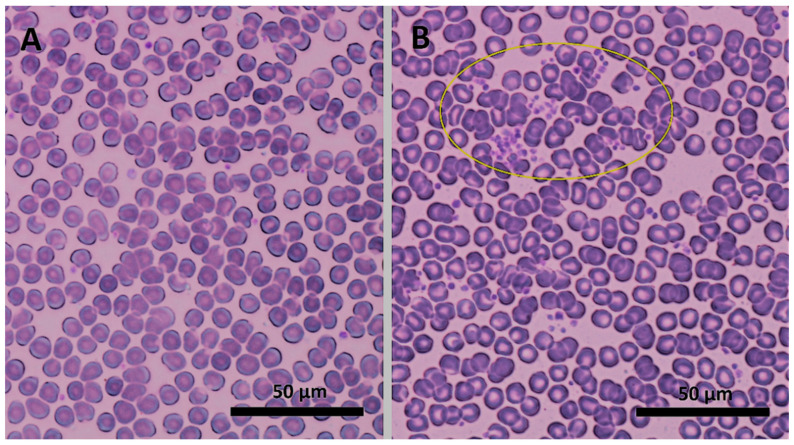
Images of blood smears before (**A**) and after (**B**) adding and incubation of the second-generation dendron D2 at a concentration of 10 µM (C2) from light microscopy at 100× magnification. The small purple particles represent platelets, which under these experimental conditions created clumps (yellow ellipse).

The investigated blood parameters were not significantly different between paired (before/after incubation) control samples. Thus, we could exclude the effect of incubation time on the observed values. No apparent hemolysis or discoloration occurred upon addition or after incubation with D1 and D2 dendrons. Analysis of the red component of blood did not reveal any clinically relevant red blood cell (RBC) aggregation at the selected concentrations C1 and C2, as evaluated by RBC count, mean cell volume, distribution width, mean corpuscular hemoglobin concentration, and hematocrit values under all combinations of experimental conditions (Table A1). This conclusion was confirmed by viewing blood smears under the light microscope. No abnormalities in size, shape, or colour of RBCs were identified (Figure 1).

**Figure 2:**
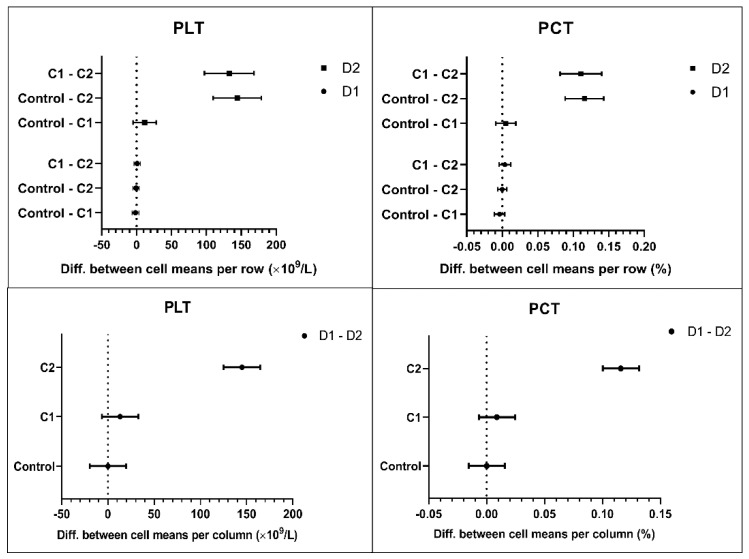
Changes in platelet’s parameters expressed as mean within-subject differences in one factor within the levels of the other factor. The upper plots show simple main effects of concentration, and the lower plots the effect of generation. The symbols denote mean responses with 95% CI. Abbreviations: PLT, absolute number of platelets; PCT, plateletcrit.

Similarly, no abnormal results were obtained for the white component of blood except, for an insignificant increase in the apparent count of leukocytes (WBCs) upon the incubation in the D2C2 experimental condition. Overestimation of WBCs can be seen in the presence of platelet clumping. The platelet clumps had the same size as small WBCs, and thus appeared as an interference at the lower threshold of an impedance WBC histogram, spuriously increasing the WBC count. The morphological analysis of platelets included the estimation of the number of platelets (PLT), plateletcrit (PCT), mean platelet volume (MPV), platelet distribution width (PDW), and several other parameters (Table A1). Upon incubation with D2C2, we found spuriously low platelet counts and lower PCT (Figure 2), likely due to formation of platelet microaggregates that could be misclassified by the hematology analyzer as leukocytes.

This notion was supported by the above-mentioned finding of slightly increased WBC counts and by no significant change in PDW. Pseudothrombocytopenia due to time/anticoagulant-induced platelet aggregation was excluded by comparing the treated samples with the respective control samples (evaluated before and after the incubation period).

**Figure 3:**
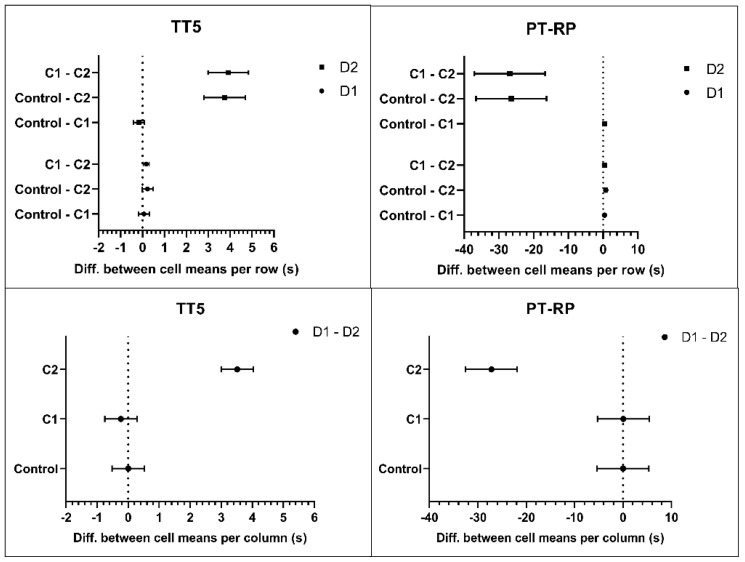
Changes in coagulation parameters expressed as differences in one factor within the levels of the other factor. The upper plots show simple main effects of concentration, and the lower plots the effect of generation. The symbols denote mean responses with 95% CI. Abbreviations: TT5, thrombin time; PT−RP, prothrombin time (RP, recombiplastine). Note: changes in APTT−SS—activated partial thromboplastine time (reagent: SynthA Sil) followed the same pattern as those for PT-RP.

The microaggregates were confirmed by viewing blood smears presenting small platelet clumps of 5–20 cells (Figure 1). Some samples showed signs of hypogranulation or degranulation, which showed that, upon incubation with D2 at C2, some platelets might have been activated and caused changes in coagulation indices TT5, PT, and APTT (Figure 3, Table A1).

Statistical analysis yielded a significant interaction (statistical interaction should not be confused with molecular and supramolecular interactions) between both experimental factors (dendron concentration × dendron generation). This meant that we cannot discuss our results in terms of independent main effects—we had to consider the ways in which the two factors were working together by looking at each of the simple main effects. The factor generation had two simple main effects: the effect of generation at lower concentration C1, and the effect of generation at higher concentration C2. There was no effect of generation at C1, but there was a large effect of generation at C2: the bigger dendron (D2) applied at the higher concentration (C2) caused changes in the platelet indices, whereas the smaller dendron (D1) did not. All simple effects in PLT indices, presented as within-subject contrasts, are shown in Figure 2 (PLT and PCT) and Figure 3 (TT5 and PT-RP). The graphs visually confirm a clear nonadditive effect of the combination of higher concentration and higher generation of the amphiphilic dendrons. The differences obtained for the rest of hematology and coagulation indices were broadly consistent across all experimental conditions and did not differ from their respective reference values.

### 3.2. Interaction of Dendrons with Model Lipid Membranes—Liposomes

**Figure 4:**
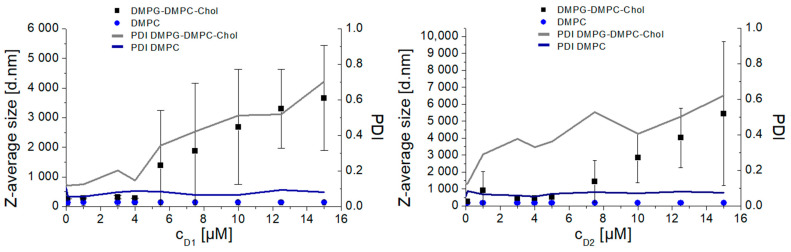
The hydrodynamic diameter (Z-average; (d.nm + SD)) and polydispersity index (PDI) of zwitterionic DMPC and negatively charged DMPG−DMPC−Chol liposomes upon the increasing concentration of D1 (**left**) and D2 (**right**) dendrons in Na-phosphate buffer (10 mM, pH 7.4) at 25 °C. Error bars represent standard deviations.

**Figure 5:**
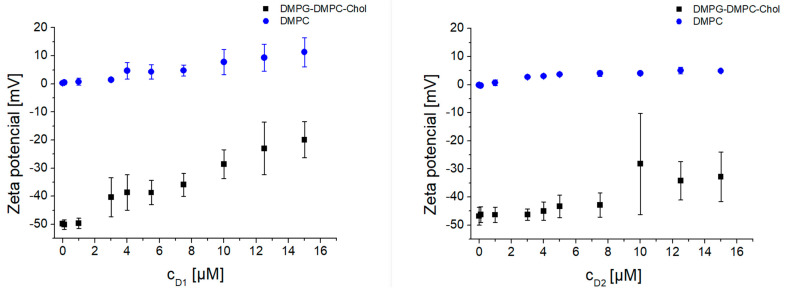
Zeta potential of zwitterionic DMPC liposomes and negatively charged DMPG−DMPC−Chol liposomes upon the increasing concentration of D1 (**left**) and D2 (**right**) dendrons in Na-phosphate buffer (10 mM, pH 7.4) at 25 °C. Error bars represent standard deviations.

**Figure A2:**
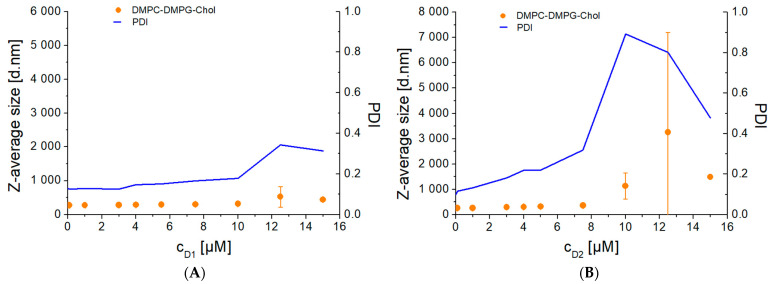
The hydrodynamic diameter (Z-average; (d.nm + SD)) and polydispersity index (PDI) of negatively charged DMPG−DMPC−Chol liposomes upon the increasing concentration of D1 (**A**) and D2 (**B**) dendrons in Na-phosphate buffer (10 mM, pH 7.4) at 37 °C.

**Figure A3:**
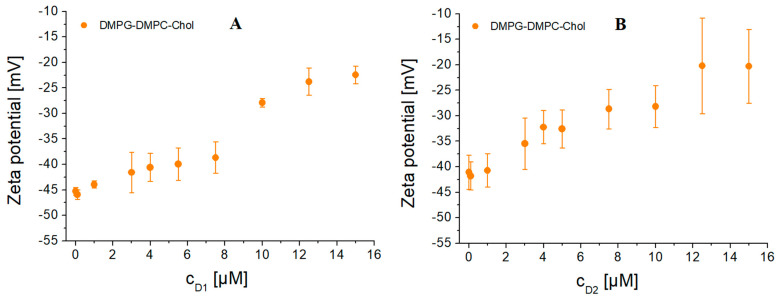
Zeta potential of negatively charged DMPG−DMPC−Chol liposomes upon the increasing concentration of D1 (**A**) and D2 (**B**) dendrons in Na-phosphate buffer (10 mM, pH 7.4) at 37 °C.

To further investigate the mechanism of interaction, experiments on the model lipid membranes were performed. In the first series of experiments, we studied the interaction of amphiphilic phosphorous dendrons D1 and D2 with DMPG-DMPC-Chol liposomes. Results were obtained by titration of the dendrons with concentrations from 0.1 µM up to 15 µM. Three independent replicates were obtained for each experimental situation in order to evaluate differences in the responses for both systems, negatively charged and zwitterionic liposomes. Each replicate data point was measured three times to increase precision, and the average was entered into the data set. We observed a significant increase in (Z-average size) from 250 nm up to more than 1 µm, with higher dendron concentrations at 25 °C (Figure 4). Similarly, we observed an increase in PDI from 0.2, a uniform distribution in particle sizes, up to 1, a highly polydisperse sample. In addition, measurements at 37 °C had later onset of aggregates for both dendrons (Appendix B, Figure A2). Zeta potential results showed an increasing pattern in correspondence to dendron concentration applied (Figure 5, Figure A3). The negative zeta potential of the liposomes titrated with D2 changed from −48.27 ± 2.61 mV up to as high as −32.8 ± 8.79 mV at 25 °C (Figure 5, right). At 37 °C, the observed increase was from −43.18 ± 3.15 mV for up to −20.3 ± 7.25 mV (Figure A3, right).

To confirm the electrostatic nature of dendron interaction with the lipid membrane, we prepared neutral DMPC liposomes. As presumed, the dendrons did not interact with the neutral DMPC liposomes in terms of change of hydrodynamic diameter. Following addition of any dendron concentration, the Z-average size was in the range of 182.21 ± 8.31 nm. Zeta potential values did not fluctuate as much as in the case of negatively charged DMPG-DMPC-Chol liposomes. The extra sum-of-squares F test and the corrected Akaike’s criterion (AICc) were used to compare the differences between the two modeled data sets at the given experimental condition. We proved that the response curves for negatively charged (DMPG−DMP−Chol) and zwitterionic liposomes (DMPC) significantly differed in both physical characteristics (*p* < 0.0001) (Appendix B).

## 4. Discussion

In vitro methods are critical components in the biological safety assessment of NP intended for medical use in humans, as they provide valuable information on cell and tissue compatibility prior to preclinical and clinical studies. While such approaches utilize simplified systems compared to the complex in vivo milieu, they are important because they provide insight into potential in vivo tissue and cellular responses. Findings of such studies can be compared to those obtained with experimental models.

To study the in vitro effect of generation and concentration on the blood components, we chose a within-subject study design that could help to reduce errors associated with individual differences. The blood samples from the same participants were subjected to both experimental and control conditions, which enabled us to adjust the observed effects to account for inter-subject variability as part of the statistical analysis. Our results for in vitro assessed blood compatibility were largely satisfying—most hematology indices did not deviate from their baseline values. We did not observe any unusual RBC aggregation or adhesiveness to other cells upon adding dendrons. No morphological alterations occurred in RBCs and WBCs as a result of their exposure to NPs. The RBCs were negatively charged, which prevented cells from coming into contact with them [18]. Thus, we could assume that the balance between the attractive and repulsive electrostatic forces between RBCs was not disturbed by the amphiphilic dendrons.

Our results showed that the best blood compatibility was achieved with the combination of lower generation and lower concentration (D1C1 experimental condition), while a lower but still satisfying compatibility was detected for the D1C2 and D2C1 conditions. Only the second-generation dendron at a final concentration of 10 µM exerted effects with potential clinical relevance. That meant the main effects of the factors generation and concentration were not additive, which might be attributed to the nonlinear increase in the number of functional end groups increasing positive charge on the nanoparticle surface. The observed platelet abnormalities could be described as signs of pseudo-thrombocytopenia (decreased PLT and PTC) and a consumption coagulopathy (shortened TT5 and prolonged PT-RP and APTT-SS). The former condition is linked to platelet clumping—as a result, reported platelet counts were lower than their actual counts in the blood, because automated counters cannot differentiate platelet clumps from individual cells. The latter could be linked to a deficiency of the plasma factors prothrombin, fibrinogen, factor V, or factor X [16,18]. Assuming that the reference range of the APTT was 30–40 s and the reference range of the PTT was 60–70 s [19], the observed out-of-range prolongation in APTT-SS was linked to the increasing number of accessible positively charged groups in the D2C2 experimental condition. These observations can be regarded as a physiological response to pathologic overstimulation of the coagulation system. Some degree of activation of coagulation may be observed in several clinical conditions, especially those associated with a systemic inflammatory response. When the procoagulant stimulus is sufficiently severe and overcomes the natural anticoagulant mechanisms of coagulation, a condition known as disseminated intravascular coagulation (DIC) may occur [20]. Our results obtained for higher-generation dendrons at higher concentrations were suggestive of an incipient coagulopathy manifested as abnormalities in routine coagulation parameters. In our measurements, which were controlled for relevant extraneous factors, these abnormalities could be attributed to the size of the NPs, and the exponential increase in surface area and the number of the functional groups. This was in line with the higher toxicity observed for cationic amino-modified NPs [12]. The impact of the surface charge of commercial PAMAM G4 dendrimers on their interactions with human serum albumin, and with alpha-1-microglobulin, were studied by Serchenya et al. [21]. Both proteins could bind and transfer various ligands in blood and have immunoreactivity properties, which could be affected by the interaction with the NPs. The authors found that the weakest interaction was observed for anionic dendrimers, and that the effect of neutral and cationic dendrimers was comparable. They confirmed that the binding of cationic dendrimers to proteins could be explained by electrostatic forces acting between positively charged dendrimers and negatively charged regions of proteins, and that the effect of cationic dendrimers on proteins was generation-dependent [21,22]. Their most important finding was that dendrimer-induced reduction in immunoreactivity of the proteins was only partial, even if the protein was fully bound by the dendrimers. Thus, the application of dendrimers in vivo might not eliminate the immunochemical properties of these proteins [22].

Based on our results obtained in in vitro assays using human blood and experiments using cell models, we assumed that the interactions between amphiphilic phosphorous dendrons and blood components were most likely of an electrostatic nature. As seen in Figure 5, the dendrons did not interact with neutral DMPC liposomes in terms of changes in their hydrodynamic diameter. In contrast, a significant interaction was observed with negatively charged DMPG-DMPC-Chol liposomes, where large aggregates were observed. PDI corresponded to the changes in size and forming aggregates. In the case of neutral DMPC liposomes, PDI values were less than 0.2 for any dendron concentration applied. On the other hand, with negatively charged liposomes, PDI reached values as high as 1. Zeta potential values increased in both experimental conditions with increasing concentrations of D1 and D2, which was consistent with the positive surface charge of the dendrons. Therefore, understanding specific relationships between the size and chemistry of dendron-based NPs, and their interactions with biological systems, is essential for assessing possible toxicity in humans and optimizing designs for in vivo use.

In spite of the fact that dendritic nanoparticles may undergo nonspecific binding to spurious targets within the living body, several types of dendrimers are commercially available, and are already being used in clinical trials and settings; e.g., Superfect^®^, Priofect^®^ (PAMAM dendrimers used as transfection agents), and Vivagel^®^ (Poly-L-Lysine dendrimers applied to prevent the transmission of HIV and sexually transmitted diseases). Some dendrimer complexes, such as dendrimer-docetaxel or dendrimer-oxaliplatin by Starpharma, are also being studied and preclinically tested as a treatment for breast and colon cancer, respectively [23].

Overall, our results obtained for blood components and for liposomes as simple cell models implied, first, a nonlinear dependence of the dendron-induced response on its concentration, and second, a range of nonobservable effect, the width of which decreased with increasing generation of the amphiphilic phosphorous dendron.

## 5. Conclusions

Our results suggested particularly good blood compatibility for lower generations of amphiphilic phosphorous dendrons reaching low concentration levels at the site of action or accidental release. However, treatment with higher-generation dendrons at higher concentrations affected several hematological and coagulation parameters in a clear concentration- and generation-dependent manner. We showed that an increase in positive surface charge reduced interparticle repulsion, which may lead to their interaction with plasma coagulation factor and/or with platelets, and eventually to platelet activation. Obtained data indicated that thrombogenic propensity might not be the only issue in nanomedicine safety. Coagulation abnormalities may also relate to prolonged clotting time.

Thus, some precautions must be taken while using higher-generation amphiphilic dendrons at higher concentrations, since interactions of the naked or prematurely released nanoparticles with platelets and clotting factors cannot be excluded.

## Data Availability

Not applicable.

## Appendix A

**Table A1:** Descriptive statistics and differences in the selected hematology and coagulation parameters across experimental conditions.

	D1				D2			
	Mean 1	Mean 2	Diff	Adjusted *p*	Mean 1	Mean 2	Diff	Adjusted *p*
**RBC (×10** **^12^** **/L)**								
Control vs. C1	4.80	4.89	−0.088	0.0001	4.80	4.84	−0.043	0.0242
Control vs. C2	4.80	4.84	−0.039	0.0567	4.80	4.82	−0.024	0.1979
C1 vs. C2	4.89	4.84	0.049	0.0383	4.84	4.82	0.019	0.3048
**HBG (g/L)**								
Control vs. C1	141.1	144.4	−3.357	<0.0001	141.1	142.2	−1.143	0.0487
Control vs. C2	141.1	143.6	−2.500	<0.0001	141.1	141.9	−0.786	0.1713
C1 vs. C2	144.4	143.6	0.857	0.1388	142.2	141.9	0.357	0.4067
**PLT (×10** **^9^** **/L)**								
Control vs. C1	225.7	227.4	−1.643	0.6465	225.7	214.2	11.50	0.1794
Control vs. C2	225.7	226.4	−0.714	0.9057	225.7	81.50	144.2	<0.0001
C1 vs. C2	227.4	226.4	0.929	0.8488	214.2	81.50	132.7	<0.0001
**MPV (fL)**								
Control vs. C1	9.31	9.41	−0.100	0.2754	9.31	9.61	−0.300	0.0275
Control vs. C2	9.31	9.34	−0.021	0.9333	9.31	11.44	−2.129	<0.0001
C1 vs. C2	9.41	9.34	0.079	0.4836	9.61	11.44	−1.829	<0.0001
**PCT (%)**								
Control vs. C1	0.209	0.213	−0.0039	0.3554	0.209	0.204	0.0050	0.6057
Control vs. C2	0.209	0.209	−0.0001	0.9995	0.209	0.094	0.1158	<0.0001
C1 vs. C2	0.213	0.209	0.0038	0.4495	0.204	0.094	0.1108	<0.0001
**PDW (%)**								
Control vs. C1	16.88	16.91	−0.029	0.879	16.88	17.11	−0.229	0.0631
Control vs. C2	16.88	16.84	0.036	0.8377	16.88	17.19	−0.307	0.1616
C1 vs. C2	16.91	16.84	0.064	0.3207	17.11	17.19	−0.079	0.8976
**TT5 (s)**								
Control vs. C1	14.37	14.31	0.064	0.7748	14.37	14.54	−0.171	0.1792
Control vs. C2	14.37	14.14	0.229	0.0650	14.37	10.63	3.743	<0.0001
C1 vs. C2	14.31	14.14	0.164	0.0216	14.54	10.63	3.914	<0.0001
**PT-RP (s)**								
Control vs. C1	11.73	11.34	0.386	0.0003	11.73	11.29	0.443	0.0082
Control vs. C2	11.73	10.96	0.764	0.0010	11.73	38.16	−26.43	<0.0001
C1 vs. C2	11.34	10.96	0.379	0.1227	11.29	38.16	−26.87	<0.0001
**aPTT-SS (s)**								
Control vs. C1	29.77	32.80	−3.029	<0.0001	29.77	50.81	−21.04	0.0006
Control vs. C2	29.77	34.18	−4.407	0.0007	29.77	152.4	−122.6	<0.0001
C1 vs. C2	32.80	34.18	−1.379	0.1690	50.81	152.4	−101.6	<0.0001
**FBG (g/L)**								
Control vs. C1	2.760	2.804	−0.044	0.6181	2.760	2.786	−0.026	0.8923
Control vs. C2	2.760	2.701	0.059	0.3756	2.760	2.829	−0.069	0.2544
C1 vs. C2	2.804	2.701	0.104	0.0375	2.786	2.829	−0.043	0.6422

## Appendix B

### Interaction of Amphiphilic Phosphorous Dendrons with Model Lipid Membranes—Liposomes—Statistical Analysis

D1 zeta-average size:

F (DFn, DFd) 17.34 (4, 51); difference in AICc 40.13; we rejected the null hypothesis and accepted the research hypothesis stating that there was a different curve for each data set (*p* < 0.0001).

D2 zeta-average size:

F (DFn, DFd) 31.51 (4, 52); difference in AICc 63.36; we rejected the null hypothesis and accepted the research hypothesis stating that there was a different curve for each data set (*p* < 0.0001).

D1 zeta potential:

F (DFn, DFd) 430.1 (4, 51); difference in AICc 198.8; we rejected the null hypothesis and accepted the research hypothesis stating that there was a different curve for each data set (*p* < 0.0001).

D2 zeta potential:

F (DFn, DFd) 307.7 (4, 52); difference in AICc 181.8; we rejected the null hypothesis and accepted the research hypothesis stating that there was a different curve for each data set (*p* < 0.0001).
